# Algorithms for Liver Segmentation in Computed Tomography Scans: A Historical Perspective

**DOI:** 10.3390/s24061752

**Published:** 2024-03-08

**Authors:** Stephanie Batista Niño, Jorge Bernardino, Inês Domingues

**Affiliations:** 1 Polytechnic Institute of Coimbra, Coimbra Institute of Engineering, Rua Pedro Nunes-Quinta da Nora, 3030-199 Coimbra, Portugal; a2019114900@isec.pt (S.B.N.); jorge@isec.pt (J.B.); 2 Centre for Informatics and Systems, University of Coimbra (CISUC), Pólo II, Pinhal de Marrocos, 3030-290 Coimbra, Portugal; 3 Medical Physics, Radiobiology and Radiological Protection Group, Research Centre of the Portuguese Institute of Oncology of Porto (CI-IPOP), 4200-072 Porto, Portugal

**Keywords:** artificial intelligence, computed tomography, hepatic pathologies, liver segmentation

## Abstract

Oncology has emerged as a crucial field of study in the domain of medicine. Computed tomography has gained widespread adoption as a radiological modality for the identification and characterisation of pathologies, particularly in oncology, enabling precise identification of affected organs and tissues. However, achieving accurate liver segmentation in computed tomography scans remains a challenge due to the presence of artefacts and the varying densities of soft tissues and adjacent organs. This paper compares artificial intelligence algorithms and traditional medical image processing techniques to assist radiologists in liver segmentation in computed tomography scans and evaluates their accuracy and efficiency. Despite notable progress in the field, the limited availability of public datasets remains a significant barrier to broad participation in research studies and replication of methodologies. Future directions should focus on increasing the accessibility of public datasets, establishing standardised evaluation metrics, and advancing the development of three-dimensional segmentation techniques. In addition, maintaining a collaborative relationship between technological advances and medical expertise is essential to ensure that these innovations not only achieve technical accuracy, but also remain aligned with clinical needs and realities. This synergy ensures their applicability and effectiveness in real-world healthcare environments.

## 1. Introduction

As one of the most important organs in the digestive system, the liver performs critical functions such as breaking down nutrients, producing bile and eliminating toxic substances. However, liver-related diseases, particularly oncological diseases, pose health risks, and liver cancer is a leading cause of cancer-related mortality worldwide [1,2]. Computed tomography (CT) has become an integral part of diagnosis, treatment planning and monitoring the progress of oncological diseases [3,4], providing detailed cross-sectional images for accurate visualisation of internal structures, including liver tumours [5,6].

With the advancement of technology and artificial intelligence (AI) in medicine, there is a growing need to optimise the identification of oncological diseases [7]. Medical image segmentation is emerging as a fundamental step in the pipeline [8,9,10,11]. Liver segmentation in CT scans has emerged as a critical area, requiring accurate identification and delimitation of the liver region for treatment planning and progress monitoring, as well as for early detection of liver lesions and metastases to other organs [12,13,14]. However, accurate liver segmentation on CT scans is challenging due to factors such as artefacts, varying soft tissue densities and the complexity caused by adjacent organ proximity [15,16].

The aim of this paper is to provide an overview of the state of the art in the application of AI, as well as traditional methods and techniques, for liver segmentation in CT scans to enable an understanding of which factors most influence the performance of the models and methods used by the selected studies and lead them to perform differently for the same objective.

Some of the aspects that will be compared include datasets considerations, algorithms used, robustness, performance and evaluation. Specifically, we aim to answer the following research questions (RQs):

- RQ1—What are the challenges and limitations associated with accurate liver segmentation in CT scans?
- RQ2—How does the choice of the method impact the accuracy and efficiency of liver segmentation in CT scans?
- RQ3—What are the evaluation metrics commonly used to assess the performance of AI models and traditional methods for liver segmentation in CT scans?

A systematic review of the literature was conducted to address these research questions. Google Scholar was used for document retrieval, and the most representative documents from each year were selected. It is worth noting that only a few studies were found that provided an intuitive and visual approach for healthcare professionals to manipulate and interpret the segmentation results.

This paper reviews studies on liver segmentation from CT scans, organised according to the methods used. The paper’s selection methodology is detailed in Section 2, followed by a categorisation of AI models and medical image processing methods in Section 3. Section 4 summarises the main findings, while Section 5 provides a discussion of the results of the methods used. The paper concludes in Section 6 with the conclusions and directions for future work.

## 2. Methodology

This section outlines the approach taken in conducting the literature review, which involved synthesising existing knowledge, critically assessing methodologies and analysing the results to compare the performance of each AI model and traditional methods for liver segmentation in CT scans.

### 2.1. Data Sources

Google Scholar (https://scholar.google.com/, accessed on 20 January 2024) has developed over the years and has become a robust database for the scientific literature [17]. It was therefore chosen as the research tool for the present study.

### 2.2. Search Queries

A search performed on 30 January 2024, with the query “intitle:Liver + intitle:segmentation + (intitle:CT OR + intitle: tomography)” returned approximately 980 results in just 0.04 s.

### 2.3. Inclusion Criteria

The most relevant papers from each year have been included in this historical overview. In the context of Google Scholar, relevance refers to the degree to which the search results match the criteria or the context of the query. The sorting algorithm takes into account several factors to determine the order of the results, including the presence of search terms and citation counts. In addition, the review papers were all included in the current state of the art, which amounted to a further five documents.

### 2.4. Exclusion Criteria

Papers that did not meet the criteria defined in Section 2.3 were not included. Papers written in languages other than English, Portuguese and Spanish were also excluded. As a final exclusion criterion, papers were excluded if the full document was not publicly available.

### 2.5. Characterisation of Selected Papers

**Figure 1:**
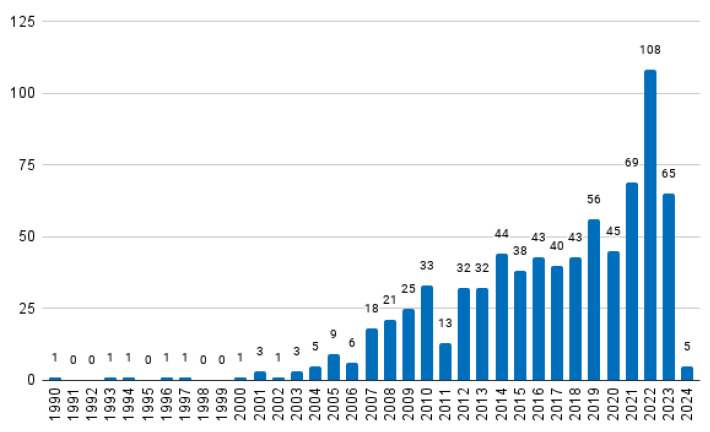
Distribution of the retrieved papers over the years.

The earliest paper dates to 1990, and thus, a potential total of 35 papers could be selected (the most relevant per year between the years of 1990 and 2024). The distribution of the total number of papers retrieved per year is given in Figure 1. As can be seen, some years do not have any papers, and thus a total of 30 documents were finally included in the historical review. It is also clear that interest in the topic has increased over the years.

**Figure 2:**
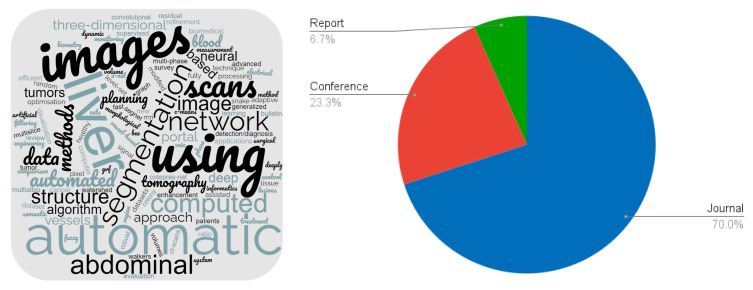
Characterisation of the selected papers.

A word cloud constructed from the titles of the selected papers is shown on the left side of Figure 2. On the right side is a pie chart showing the number of journals, conference proceedings and reports.

**Figure 3:**
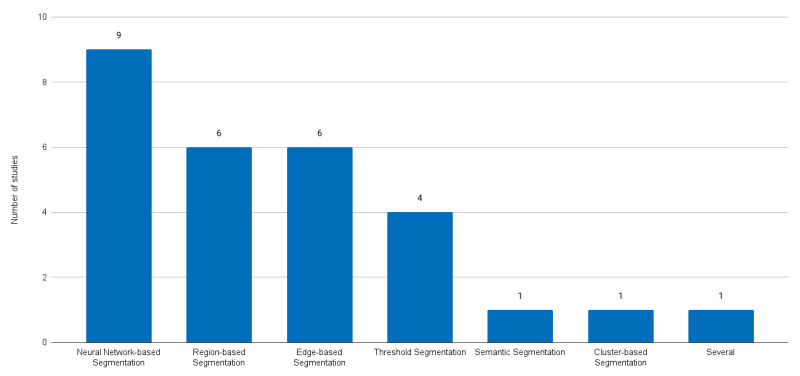
Selected paper distribution according to the categories of image segmentation techniques.

Several taxonomies can be used to characterise the reviewed papers. We decided to adopt the taxonomy introduced in a recent paper by Sakshi and Kukreja (2023) [18], published in the reputable journal Archives of Computational Methods in Engineering (Impact Factor of 9.7). Figure 3 shows the selected papers categorised by image segmentation technique according to this taxonomy.

## 3. Literature Review

Based on the selected papers, this literature review provides a historical overview of different methods and approaches for liver segmentation in medical imaging (Section 3.1). It covers neuronal-network-based segmentation, region-based segmentation, edge-based segmentation, threshold segmentation, semantic segmentation and cluster-based segmentation techniques. This review includes a comparison of the results and discusses the potential applications and strengths of each method. Section 3.2 compares this review with other existing summaries of the state of the art.

### 3.1. Historical Overview

As mentioned in Section 2, one paper per year between the years of 1990 and 2024 was selected, summing to a total of 35 documents. For five years, the search retrieved no results (see Figure 1), resulting in 30 documents. Unfortunately, the full texts of two works were not available. The remaining 28 documents are briefly described below.

The oldest work found to tackle liver segmentation is the one by Bae et al. (1993) [19], presenting a similar sequential image-by-image segmentation technique using a reference image, where the liver occupies a significant portion of the abdomen cross-section. Image processing techniques, including grey-level thresholding, Gaussian smoothing and connectivity tracking, are employed to extract the liver boundaries. The resulting boundaries are then smoothed using mathematical morphology techniques and B-splines. This study focuses on a living-donor liver transplant program, and the computer-determined boundaries are compared with those drawn by a radiologist, showing agreement within 10% of the calculated areas.

Gao et al. (1996) [20] focus on facilitating 3D visualisations for surgical planning. Their method employs a global histogram analysis, morphologic operations and a parametrically deformable contour model to delineate the liver boundary. Ten cases were used to validate the approach and promising results were found with minimal operator intervention required.

Soler et al. (1997) [21] propose an automatic method for segmenting the portal vein, with the primary objective of achieving accurate segmentation with detailed branching and topological information, facilitating the localisation of liver tumours concerning Couinaud’s anatomical segmentation. This approach involves the initial detection of liver contours using 3D deformable models, followed by limiting the CT images to a liver mask containing hepatic tissue, vascular trees and potential tumours. Classification of anatomical structures is performed using Gaussian curves fitted to an intensity histogram. The vascular trees and tumours are segmented through a hysteresis thresholding technique based on a distance map, considering the Gaussian parameters. An isotropic image is obtained through shape-based interpolation and the portal vein is reconstructed using skeletonization, eliminating short branches and correcting errors. Results demonstrate that the algorithm automatically extracts the first three main bifurcations of the portal vein, comparable to manual segmentation.

Yoo et al. (2000) [22] focus on the use of pixel ratios. By analysing the grey value range of a normal liver in CT images, a binary image is generated and then processed into four mesh images based on hole ratios to eliminate noise. A template representing the general outline of the liver is generated from the union image of these mesh images and subtracted from the binary image to accurately represent the organ boundary. The pixel ratio, which takes into account the distribution of organ pixels, was used to discriminate between the organ and noise, especially in cases where organs have similar grey value ranges. The proposed method reduced the processing time compared to existing methods and was validated against manual segmentation by medical experts.

Pan and Dawant (2001) [23] introduce a level-set approach, which addresses the challenge of defining appropriate speed functions for contour propagation. A speed function is proposed to stop the propagation of the contour at organ boundaries with weak edges by incorporating the accumulative speed based on the path of the contour, enhancing the robustness of segmentation in noisy images. The method also leverages a priori anatomical information to improve the accuracy. Tested on five CT datasets, including cases with abnormal livers, tise method demonstrates good agreement with manual delineations.

Saitoh et al. (2002) [24] present an automated method for segmenting the liver region from the third phase of abdominal CT scans. Their approach involves the extraction of blood vessels using a threshold, followed by morphological dilation to define an approximate liver region useful for the removal of adjacent organs. The final liver region is then extracted using a threshold. The method is thus based on mathematical morphology and thresholding techniques, using the unique characteristics of blood vessels to functionally identify the liver region. The experiments performed on eight CT datasets show a good agreement between the automatically and manually detected liver regions.

Masumoto et al. (2003) [25] use multislice CT images. Their method uses two time-varying images acquired during the contrast medium circulation phase, highlighting the liver region through CT value changes. The proposed scheme involves generating a liver likelihood image by analysing CT value changes and subsequently extracting the liver region while considering the geometric characteristics of blood vessels and tumours. The evaluation, based on receiver operating characteristic (ROC) analyses, demonstrates the superiority of the proposed method over other approaches, especially when using information from both phases.

The scheme proposed by Lim et al. (2004) [26] uses an ROI approach to optimise computational efficiency. Morphological filters, incorporating a priori knowledge of liver location and intensity, detect the initial boundary. The algorithm then generates a gradient image using the weighted initial boundary and employs an immersion-based watershed algorithm for segmentation. Post-processing includes region merging based on statistical information to refine the segmentation.

Liu et al. (2005) [27] present a gradient vector flow (GVF) snake-based method for the semi-automatic segmentation of liver volumes in contrast-enhanced CT images. The algorithm follows a stepwise approach, starting with the computation of an initial edge map using the Canny edge detector and the estimation of a liver template. The edge map is then modified to suppress edges within the liver using the liver template, and a concavity removal algorithm is applied to refine the liver boundary. The GVF field is computed based on the modified edge map, and the initial liver contour is determined by considering the candidate initial contour and the computed GVF field. The final liver contour is obtained by deforming the initial contour using the snake. The method was evaluated on 20 contrast-enhanced volumetric liver images, and the results were compared with a radiologist’s manual delineation. The median difference ratio between the computer-generated results and manual results is 5.3%, with a range of 2.9% to 7.6%.

A three-stage approach is used by Lim et al. (2006) [28]. The first stage involves image simplification as preprocessing, where an ROI is identified and thresholds are determined using multilevel thresholding. The second stage detects a search range using multiscale morphological filtering, region labelling, and partition clustering. The third stage uses a contour-based segmentation approach with a labelling-based search algorithm to refine the initial liver boundary. The effectiveness of the algorithm is demonstrated through experimental results on contrast-enhanced abdominal CT images, with an average segmentation accuracy of 96%. Volume measurement is performed based on the segmented liver regions, with an average error rate of 3%.

Beichel et al. (2007) [29] introduce a two-step process. First, initial segmentation is performed using graph cuts, overcoming challenges such as the high variability in liver shape and grey-value appearance. Second, an interactive refinement step is introduced, allowing users to correct segmentation errors in a 3D environment. The refinement is facilitated by a hybrid desktop/virtual reality (VR) user interface. This approach is demonstrated on ten contrast-enhanced liver CT scans, demonstrating robustness to variations in patient data. The results also indicate an improved segmentation quality with low interaction times.

Massoptier and Casciaro (2008) [30] present a fully automated method that uses a statistical-model-based approach to distinguish liver tissue from other abdominal organs. An active contour technique using gradient vector flow is used for smoother segmentation of the liver surface. Automatic classification is performed to isolate hepatic lesions from liver parenchyma. The method was evaluated on 21 datasets and demonstrated robust and efficient liver and lesion segmentations close to the ground truth, with an average processing time of 11.4 s per 512 × 512 pixel slice. The volume overlap for liver surface segmentation is 94.2%, and the accuracy is 3.7 mm. Tumour detection achieved a sensitivity and specificity of 82.6% and 87.5%, respectively.

Heimann et al. (2009) [31] focus on the comparison and evaluation of different methods. The image data, acquired from different CT scanners, consisted of contrast-dye-enhanced scans showing pathological conditions like tumours and cysts. Radiology experts manually delineated the liver contours in transversal slices to create reference segmentations. A total of 40 images were divided into training and test sets for algorithm evaluation. Evaluation measures included volumetric overlap, relative volume difference, and surface distances. Fully automated and interactive segmentation methods were employed, with the former showing discernible performance differences. The best-performing automated approaches used statistical shape models. Interactive methods achieved higher scores with more user interaction. A combined approach using majority voting from the best-performing methods outperformed individual automated and interactive results.

A three-step procedure is outlined by Akram et al. (2010) [32]. Firstly, a pre-processing step involves converting the image to greyscale and applying a 3 × 3 median filter to reduce noise. The second step focuses on liver segmentation, with a global threshold and morphological operations to obtain the final segmented liver region. Finally, post-processing steps include adaptive histogram equalisation, Gaussian smoothing, and grey-level transformations to enhance the segmented liver region. Experimental tests on 100 CT images demonstrate the accuracy of the proposed method by comparing automated segmentation results with images manually segmented by hepatologists and oncologists.

The approach of Oliveira et al. (2011) [33] involves a sequence of four steps. First, the liver is segmented using level sets with parameters optimised by a genetic algorithm (GA). A Gaussian fit is employed to define the speed image for level set propagation. Secondly, vessels and nodules are segmented using a Gaussian mixture model, focusing on adipose nodules. A region-growing method with information from the Gaussian model is applied. Thirdly, vessels are classified into portal veins or hepatic veins using a vein tracking method. Finally, a geometric approach based on the identified veins is used to segment the liver into different Couinaud regions. Liver segmentation is based on the assumption that the liver parenchyma homogeneity and veins are mainly inside the liver. The parameters are estimated using a GA, and the fitness evaluation involves comparing the segmentation with a reference using five disparity metrics. The proposed method shows good performance, ranking among the top methods in the MICCAI-SLiver07 conference evaluation.

The method developed by Linguraru et al. (2012) [34] uses a robust parametrisation of 3D surfaces for point-to-point correspondence, overcoming challenges such as inconsistent contrast enhancements and imaging artefacts. A shape descriptor that is invariant under rotation and scale is used to compare the local shape features of organs. Initial liver segmentation is refined using a shape-driven geodesic active contour, and hepatic tumours are detected and segmented using graph cuts and support vector machines (SVMs). This technique is evaluated on a dataset of 101 CT scans and shows improvements in the liver segmentation accuracy, particularly in cases with large tumours and segmentation errors. Furthermore, the method identifies liver tumours with a low rate of false positives.

Li et al. (2013) [35] discuss a method that makes use of fuzzy clustering and level set techniques. The Fuzzy C-Means (FCM) clustering algorithm is employed, which assigns pixels to different categories based on fuzzy memberships, considering both the grey level intensity and spatial information. The FCM algorithm is iteratively optimised by minimising a cost function, allowing the fuzziness of the resulting partition. To overcome the limitations of standard FCM, a spatial FCM algorithm is introduced that incorporates spatial information into fuzzy membership functions. This paper also introduces the level set method, a continuous deformable model for segmentation. Distance-Regularized Level Set Evolution (DRLSE) is proposed to address reinitialisation issues and improve the efficiency. The proposed method is evaluated using accuracy, sensitivity, and specificity metrics and demonstrates a high performance in liver segmentation, especially in cases with unclear boundaries. A comprehensive review of abdominal image segmentation using soft and hard computing approaches is provided in [36].

Platero et al. (2014) [37] integrate a multi-atlas segmentation approach with graph cuts. Their method includes several steps: (1) obtaining an initial solution using low-level operations to define the ROI around the liver; (2) constructing a fast probabilistic atlas for the ROI and computing a coarse binary segmentation using segmentation-affine registration; (3) ranking the atlases based on segmentation similarity and propagating selected atlases to the target image; (4) improving the segmentation accuracy through label fusion, minimising the discrete energy function; and (5) evaluating the approach using a public liver segmentation database. The experimental results show a high accuracy, competitive with human expert segmentation.

Artificial Bee Colony (ABC) optimisation is used by Mostafa et al. (2015) [38]. Their algorithm use ABC optimisation to cluster different intensity values in abdominal CT images, followed by mathematical morphological operations to manipulate and separate the clusters. This process eliminates small and thin regions, such as flesh regions or organ edges. The extracted regions form an initial estimate of the liver area, which is further enhanced using a region-growing technique. The proposed approach demonstrates a segmentation accuracy of 93.73% on a test dataset of 38 CT images, taken in the pre-contrast phase.

A 3D deeply supervised network (DSN) is introduced by Dou et al. (2016) [39]. The proposed architecture consists of 11 layers, including 6 convolutional layers, 2 max-pooling layers, 2 deconvolution layers, and 1 softmax layer. The network is designed in a 3D format to effectively capture spatial information. The 3D DSN employs deep supervision via additional deconvolutional layers to counteract vanishing gradients, thus improving the training process. The learning objective is to minimise per-voxel-wise binary classification errors, with deep supervision injected at specific layers. The MICCAI-SLiver07 dataset is used for evaluation, demonstrating that the 3D DSN has a faster convergence and lower errors when compared to traditional 3D convolutional neuronal networks (CNNs).

Christ et al. (2017) [14] propose a cascaded fully CNN (CFCN) on CT slices that sequentially segments the liver and lesions. First, various preprocessing steps, including Hounsfield unit windowing and contrast enhancement, are applied. Then, the cascaded approach involving two U-Net architectures is used for liver and lesion segmentation. Finally, 3D conditional random fields (CRFs) are used to refine the segmentation results. Generalisation and scalability to different modalities and real-life datasets, including a diffusion-weighted magnetic resonance imaging (MRI) dataset and a large multi-centre CT dataset, are shown.

Hiraman (2018) [40] presents a slice alignment method that addresses the challenges through optimal threshold selection, skeletonization, and enhanced correlation coefficient (ECC) alignment. Next, a CNN-based liver region of interest detection method is proposed to classify 2D slices for focused processing.

The study presented by Wang et al. (2019) [41] investigates the application of a generalised CNN for automated liver segmentation and biometry using cross-sectional data from abdominal CT and MRI scans. Their retrospective study included a sample of 563 abdominal scans from 530 adults, covering different imaging modalities. The CNN was initially trained on 300 unenhanced multiecho 2D SPGR MRI sets and then subjected to transfer learning for generalisation across different imaging methods. The accuracy of the CNN was evaluated using internal and external validation datasets. This study also investigates the impact of training data size on the segmentation accuracy and explores the feasibility of using automated liver segmentation for volumetry and hepatic PDFF quantification.

Almotairi et al. (2020) [42] explore the application of the SegNet architecture. The proposed modified SegNet model uses the VGG-16 network as an encoder. Tests were performed on a standard dataset for liver CT scans (3D-IRCADb01 [43]), achieving a tumour accuracy of up to 99.9% in the training phase and 86% for tumour identification.

Ayalew et al. (2021) [44] present a modified U-Net architecture and introduce a new class balancing method. To address the class imbalance between the liver and tumours, a weighting factor is applied and slices without a tumour are removed during data preparation. The U-Net-based network architecture includes batch normalisation, dropout layers, and filter size reduction. Training involves tuning hyperparameters, such as the learning rate and batch size. The datasets used are derived from the 3D-IRCADb01 [43] and LiTS [45] databases and the results achieve a Dice Similarity Coefficient (DSC) of 0.96 and 0.74, respectively. The algorithm also introduces a novel approach for direct tumour segmentation from abdominal CT scan images, with a comparable performance to existing two-step methods.

The study of Scicluna (2022) [46] is motivated by challenges such as the Combined Healthy Abdominal Organ Segmentation (CHAOS) Challenge [47], which focuses on healthy abdominal organs. The study focuses on replicating the v16pUNet1.1C model, which demonstrated a superior performance in Task 2 of the CHAOS Challenge. Results from the v16pUNet1.1C model are presented and compared with variations in the loss function and scaling transformation. The application of a 3D largest-connected-component filter is discussed, showing improvements in mean scores.

A deep semantic segmentation CNN is used by Ezzat et al. (2023) [48]. A three-stage architecture is proposed, including pre-processing with data augmentation, deep CNN training, and testing. The CNN-based semantic segmentation model is shown to be robust, achieving a test accuracy of 98.8%. The approach does not require user input, making it accessible to non-experts.

Shao et al. (2024) [49] present the Attention Connect Network (AC-Net) for liver tumour segmentation in CT and MRI images. The AC-Net consists of two main modules: an axial attention module (AAM) and a Vision Transformer module (VTM). The AAM uses an axial attention mechanism to merge features of matching dimensions, maximising the use of spatial features extracted by a CNN. The VTM processes high-level semantic features extracted by the CNN using a methodology similar to Vision Transformers (ViTs) [50]. The network achieves a DSC of 0.90, a Jaccard coefficient (JC) of 0.82, a recall of 0.92, a precision of 0.89, a Hausdorff distance (HD) of 11.96, and an average symmetric surface distance (ASSD) of 4.59.

### 3.2. Other Review Papers

The search described in Section 2 retrieved six literature review documents. For one of the works, however, the full document was not available. The remaining five are briefly presented in the following.

A comparative analysis of various available techniques, focusing on their advantages and disadvantages, is given in [51]. Recognising the challenges posed by the variable shape of the liver and the weak edges in adjacent organ regions, this survey covers approaches such as threshold, model, level set, region, active contour, and clustering. This paper also divides its investigation into sections, covering both image pre-processing and segmentation techniques, providing an overview of the current landscape in liver segmentation from CT images.

Study [52] provides a survey of 3D image segmentation methods, focusing on selected binarization and segmentation techniques suitable for processing volume images. For thresholding methods, both global and local techniques are considered, and challenges such as hysteresis in dealing with voxel value distributions are addressed. The region growing section explores voxel-based procedures, including growing by grey value and adaptive region growing. In addition, deformable surfaces and level set methods are discussed, before other segmentation concepts such as fuzzy connectedness and watershed algorithms are introduced. The concluding remarks underline the complexity of image segmentation, emphasising the absence of a universal solution and the need to carefully evaluate and select methods based on specific tasks and dataset characteristics. The challenges posed by 3D data, including the data volume and issues of interactivity and visualisation, are also acknowledged.

Study [53] reviews the literature on methods for segmenting liver images, distinguishing between semi-automatic and fully automated techniques. The challenges of liver image segmentation, such as low contrast, blurred edges, and the complexity of the liver morphology, are discussed. Different approaches are reviewed, including neuronal-network-based methods, support-vector-machine-based methods, clustering-based methods, and hybrid methods. It is concluded that, despite progress, liver image segmentation remains a challenging task, and the authors encourage further development of hybrid approaches for more accurate segmentation.

Various segmentation methods, including statistical shape models, probabilistic atlas-based approaches, geometric deformable models, and machine-learning-based methods, are reviewed in [54]. This review includes information on avaliable databases and challenges in liver tumour segmentation, highlighting the scarcity of public datasets and the need for improved segmentation methods. Liver blood vessel segmentation and computer-assisted diagnosis (CAD) systems are also reviewed. The conclusion highlights the importance of segmentation, particularly in pathological cases, and the need for improved CAD systems with accurate segmentation for comprehensive analysis of liver treatment.

The survey paper [55] provides a comparative analysis of various available techniques, focusing on their advantages and disadvantages. Grey-level-based techniques, such as region growing and active contour methods, are highlighted as effective for liver segmentation. This survey acknowledges the challenges of detecting early-phase liver lesions and emphasises the need for a combination of methods to achieve seamless segmentation, with region growing and active contour methods considered more efficient than other segmentation techniques.

This survey differs from the other documents in this section in a number of ways. Firstly, the most recent of the review papers found dates to 2022. One of the contributions of this work is to present a more up-to-date view of the works published since then. In addition, none of the other works present a historical perspective on the subject, starting from 1990, as is the case in the present review.

## 4. Findings

**Figure 4:**
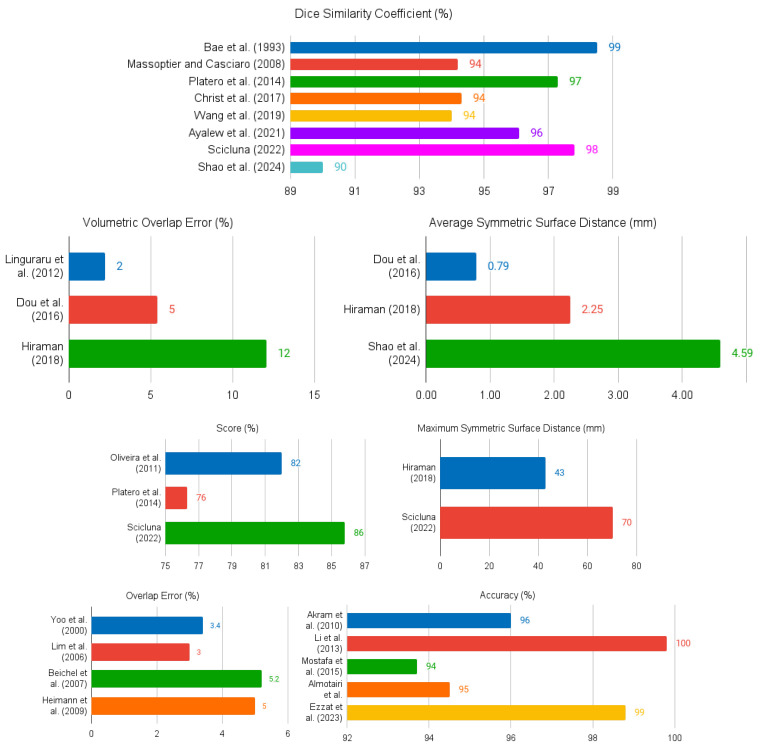
Summary of the quantitative results presented in the reviewed documents.

A summary of the papers reviewed in Section 3.1 is given in Table 1. The columns of the table contain the following information: identification of each study (column Authors); publication year (column Year); general category or approach used in the segmentation method as defined in [18] (column Segmentation Category); the specific segmentation technique or algorithm (column Method); whether the method is fully-automatic or semi-automatic (column Autom. Level for Automation Level); whether the segmentation is performed in 2D or 3D (column Dim. for Dimensionality); the dataset or database used for evaluation (column Database); and the key results of the best segmentation method in each paper (column Results). Graphical depictions of the metrics used in one or more studies are shown in Figure 4.

Table 2 presents a brief description of each segmentation category, highlights its main advantages and disadvantages, and presents some application details. It is clear from Table 1 that prior to 2016 there was no predominant category. Region-based segmentation, edge-based segmentation, threshold segmentation, semantic segmentation, cluster-based segmentation, and even combinations of several methods were tried. However, since 2016, neuronal-network-based techniques have dominated the field. Remembering the huge impact that AlexNet [56] had in winning the ImageNet Large Scale Visual Recognition Challenge (ILSVRC) in 2012 [57], it is clear that liver segmentation in CT scans took a few years to catch up with the state-of-the-art research. This is probably due to the arrival of U-Net [58], which was proposed in 2015, that is specifically designed for biomedical image segmentation.

**Figure 5:**
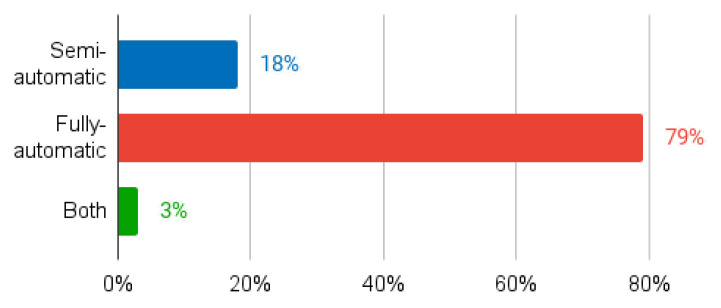
Percentage of studies according to their level of automation (semi-automatic, fully automatic, or both).

In terms of automation, both fully automated and semi-automated techniques have been explored (Figure 5). While it is nice to have fully-automatic, accurate and fast techniques, the final decision should always belong to the specialist. Thus, we advocate fully automatic methods for contour initialisation, together with the development of intuitive tools that allow specialists to modify the fully automatically generated contour if they feel the need to do so. This point of view is in line with current clinical practice, where the specialist follows contouring guidelines from respected entities such as the European Society for Radiotherapy and Oncology, the American Society for Radiation Oncology, or the Global Harmonization Group [59], while being allowed to use built-in auto-delineation and interpolation tools [60]. We therefore agree with Sarria et al. [60] in that, while AI can improve the accuracy and consistency of contouring, it cannot replace the knowledge and clinical judgement of radiation oncologists, physicists, or radiation therapists. AI should be used as a tool to support and optimise clinical decision making and not as a substitute for human expertise.

**Figure 6:**
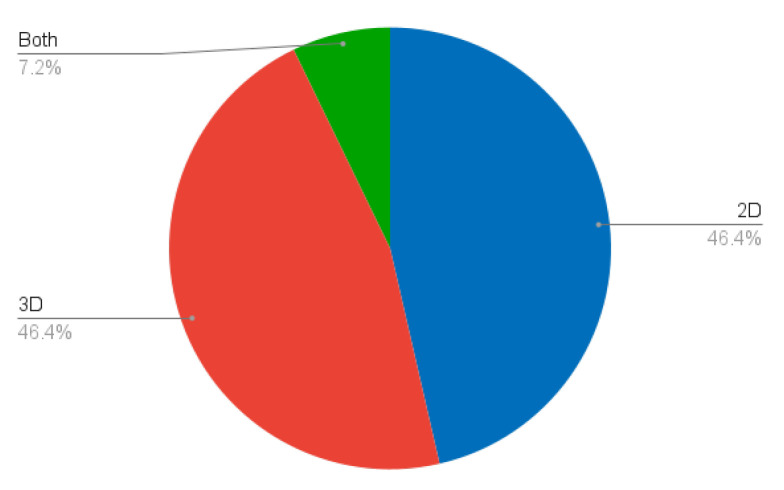
Percentage of studies according to their image dimensionality (2D vs. 3D).

Both slice-based (2D) and volume-based (3D) methods have been developed (Figure 6). As an anatomical structure, we believe that liver segmentation methods should be inherently three-dimensional. This would have two main advantages. On the one hand, fully 3D methods would make use of more contextual information and thus potentially provide better segmentations. On the other hand, the development of fully three-dimensional techniques would avoid the need for slice-based methods to aggregate all of the segmentations into a coherent volume. This aggregation could not only lead to errors and anatomically incorrect structures, but would also increase the computational time. Although we do advocate for true three-dimensional methods, we recognise their drawbacks, notably the increased complexity of implementation, the need for more computational resources, the possibly lower efficiency, the increased data requirements, and difficulties in visualisation. The choice must be an informed one, and made on a case-by-case basis.

**Figure 7:**
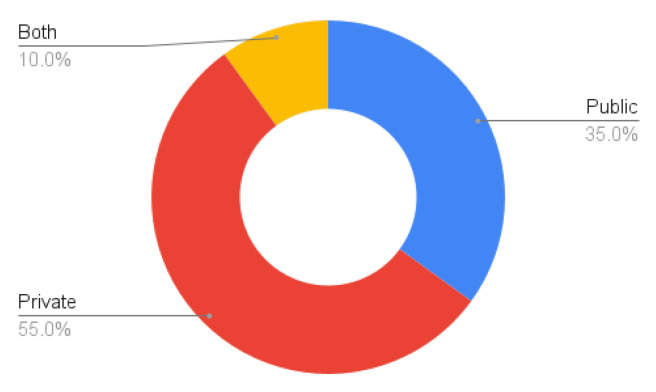
Percentage of database types.

Data availability is a major concern, as most methods use private datasets (Figure 7). This inhibits reproducibility of the results. In addition, researchers who do not have access to hospitals or other facilities with CT scanners cannot develop new techniques for this particular problem. We advocate making the data available, while respecting all the ethical considerations that are important when dealing with medical data and properly anonymising any sensitive information. Some notable exceptions to publicly available datasets are listed in Section 5.1.

## 5. Discussion

As seen in the previous section, significant advances in liver segmentation techniques have been presented, with particular impact due to the adoption of AI methods, more specifically neuronal network techniques. In this section, we provide some considerations of publicly available databases (Section 5.1), the impact of the widespread adoption of neuronal networks since 2017 (Section 5.2), a comparison between 2D and 3D implementations (Section 5.3), and Section 5.4 present answers to the research questions posed in the introductory section.

### 5.1. Public Dataset Analysis

**Figure 8:**
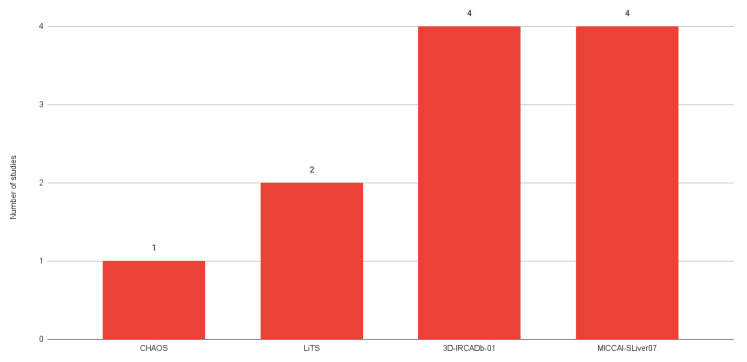
Number of studies using each public dataset.

The most common public datasets used in studies of liver segmentation on CT scans include 3D-IRCADb01, LiTS17, and MICCAI-SLiver07 (Figure 8). According to a dataset comparison provided by Al-Saeed et al. [61] (shown in Table 3), it is possible to identify several key differences that may have implications for data processing and analysis, such as different formats and differences in resolution between datasets which may require different approaches to processing and interpretation.

### 5.2. Impact of the Adoption of Neuronal-Network-Based Methods

The growth of neuronal-network-based approaches has led to remarkable progress in liver segmentation, particularly with respect to CT scans. These models have led to a new era of accuracy and efficiency, significantly outperforming traditional methods [56,58]. This improved accuracy has become critical in the field of medical imaging, where the correct interpretation of CT scans can directly affect the diagnosis and treatment plans of patients. Furthermore, the efficiency of these neuronal network models translates into faster processing times, allowing for more agile decision making in clinical settings.

Another positive aspect of neuronal networks in liver segmentation is their ability to cope with the complexity of the liver anatomy. Neuronal networks, with their systematic and complex pattern recognition capabilities, are able to navigate these anatomical variations. As a result, they are better able to deal with the variety of appearances that liver tissue can have on CT scans. This ability to handle complex datasets ensures that neuronal networks can provide consistent and accurate segmentation in a wide range of cases.

### 5.3. Comparison between 2D and 3D Methods for Liver Segmentation

- Importance of Choosing between 2D and 3D Methods
  - In medical imaging, and in particular liver segmentation, the choice between slice-based 2D and volume-based 3D segmentation methods is crucial. This decision is highly dependent on the anatomical structure of the liver. Given the complex, three-dimensional nature of the liver, 3D segmentation techniques often prove to be the most appropriate choice [21,40]. These methods are inherently designed to understand and process the volumetric characteristics of the liver, which is a critical consideration for accurate segmentation results.
- Two-Dimensional Segmentation Limitations
  - Although 2D slice-based segmentation is widely used, it has limitations, particularly when it comes to dealing with complex organs such as the liver. The main challenge with 2D methods is their inability to fully capture all the regions of the liver. They involve working with individual slices, which can provide a fragmented understanding of the organ structure, but this fragmentation can lead to inconsistencies and errors when these individual slices are aggregated to form a complete image [41].
- Three-Dimensional Segmentation Advantages
  - In order to overcome the limitations of 2D segmentation, 3D segmentation has the ability to use more contextual information. Unlike 2D methods, which visualise the liver in individual slices, 3D techniques consider the organ as a whole, as they have the ability to ensure anatomical correctness by processing the liver as a single, continuous volume, avoiding errors that can arise from the aggregation of 2D slices [14,39]. In 2D segmentation, inconsistencies can occur when individual slices are combined, leading to inaccuracies in the representation of the liver anatomy. The holistic view provided by 3D segmentation results in more accurate segmentation, as it takes into account the spatial relationships and continuity between the different sections of the liver. The inclusion of this additional contextual information can potentially lead to segmentation results, especially in complex cases where the shape and size of the liver can vary considerably.

### 5.4. Exploring Research Questions

Following the specific analysis of the studies presented and their main findings, the questions raised in Section 1 are answered as follows.

- RQ1—What are the challenges and limitations associated with accurate liver segmentation in CT scans?
  - The challenges and limitations associated with accurate liver segmentation in CT images include under-segmentation, over-segmentation, low contrast, poor boundary detection, and background segmentation due to noise. In addition, liver segmentation in CT scans is further challenged by the presence of artefacts, such as partial volumes, noise, and low sharpness and contrast between organs, making it difficult to identify the boundaries between different tissues.
- RQ2—How does the choice of the method impact the accuracy and efficiency of liver segmentation in CT scans?
  - The choice of the method has an important impact on the accuracy and efficiency of liver segmentation in CT scans. Traditional techniques such as image processing and region growing approaches have shown varying degrees of sensitivity and specificity, with some challenges in dealing with large injuries. In contrast, newer methods such as FCN and DBN-DNN and techniques like ResU-Net and SegNet showed a higher accuracy, with some reaching the highest accuracy levels. Notably, the use of GPUs has reduced processing times, thus contributing towards more efficient and accurate liver segmentation methods.
- RQ3—What are the evaluation metrics commonly used to assess the performance of AI models and traditional methods for liver segmentation in CT scans?
  - Some of the key metrics used to measure the outcome of segmentation techniques include the Dice Similarity Coefficient (DSC), accuracy, precision, sensitivity, specificity, and the segmentation speed. There is not much consistency in the metrics presented by the various studies except for the DSC.

## 6. Conclusions and Future Work

The evolution of liver segmentation techniques throughout history reflects the broad impact of AI technologies across a wide range of disciplines. The transition to fully automated segmentation methods has been an important breakthrough in the process, although the indispensable involvement of medical experts continues to play a key role in ensuring the accuracy and clinical relevance of these techniques. The emerging prevalence of 3D segmentation methods, which follow the structure of the liver, promises more accurate and anatomically consistent results.

However, there are a number of challenges that need to be addressed in order to advance the field. The lack of public datasets is one of the main barriers to the advancement of liver segmentation technologies. Research has mainly been conducted on private datasets, often restricted to specific medical centres, which limits wider participation in research and makes it difficult to replicate.

In terms of future developments, the outlook for the evolution of liver segmentation is unfolding in several core areas that promise to impact research and application in this field. The increasing availability of public datasets is key to fostering innovation, enabling the contribution of researchers from diverse backgrounds and promoting a dynamic research environment. In addition, the definition of specific standardised evaluation metrics is crucial to allow meaningful comparisons between segmentation methods and to guide the development towards more efficient, accurate, and user-friendly solutions, such as contour- and region-based metrics, performance metrics, and user intervention metrics.

The further application of 3D segmentation techniques may be a good investment, as they can provide more anatomically accurate and consistent anatomical results, overcoming the limitations of 2D segmentation. Furthermore, the effective integration of medical expertise in segmentation automation is indeed essential, with the aim of developing interfaces that allow specialists to interact with automated segmentation results, ensuring that liver segmentation tools are both technically advanced and clinically relevant and feasible. Collaboration between cutting-edge technology and human expertise is a good approach, combining the efficiency of automation with the refined understanding of healthcare professionals, whose oversight remains critical to maintain the accuracy and reliability of the final results.

## Figures and Tables

**Table 1:** Summary of reviewed documents.

Authors	Year	Segmentation Category	Method	Autom. Level	Dim.	Database	Results
Bae et al. [19]	1993	Threshold	Grey-level thresholding	Semi	2D	Private	0.985 DSC with mean percent error within 10%.
Gao et al. [20]	1996	Edge	Parametrically deformable contour model	Fully	3D	Private	13.2% of the results required user modifications.
Soler et al. [21]	1997	Edge	Deformable models	Fully	3D	Private	Claimed to be comparable to manual segmentations.
Yoo et al. [22]	2000	Threshold	Threshold	Fully	2D	Private	3.41% error.
Pan and Dawant [23]	2001	Edge	Level sets	Fully	Both	Private	[0.874, 0.963] average similarities.
Saitoh et al. [24]	2002	Threshold	Threshold	Fully	3D	Private	∼20 min computation time.
Masumoto et al. [25]	2003	Region	Differences between time-phase images	Fully	3D	Private	67% volume ratio average; 32% in the worst cases.
Lim et al. [26]	2004	Region	Watershed	Fully	2D	Private	Only qualitative.
Liu et al. [27]	2005	Edge	GVF snake	Semi	2D	Private	5.3% median value of the difference ratios.
Lim et al. [28]	2006	Semantic	Labeling-based search	Fully	2D	Private	96% average correctness; 3% average error rate.
Beichel et al. [29]	2007	Region	Graph cuts	Semi	3D	Private	5.2% average overlap error.
Massoptier and Casciaro [30]	2008	Edge	Active contour	Fully	3D	Private	94.2% mean DSC.
Heimann et al. [31]	2009	Several	Majority Voting	Both	Both	Private	5% overlap error; −0.7 volume difference; 0.8 average distance; 1.7 RMS distance; 19.1 max distance.
Akram et al. [32]	2010	Threshold	Global Threshold	Fully	3D	Private	0.96 average accuracy; 0.0017 std; 96% accurately segmented; 4% poorly segmented.
Oliveira et al. [33]	2011	Edge	Level sets	Semi	2D	SLiver07	82.05 overall score.
Linguraru et al. [34]	2012	Region	Graph cuts	Fully	3D	Private; SLiver07	2.2 VOE.
Li et al. [35]	2013	Edge	Fuzzy clustering and level set	Fully	2D	Private	0.9986 average accuracy; 0.9989 average specificity.
Platero et al. [37]	2014	Region	Graph cuts	Semi	3D	SLiver07	76.3 maximum score; 0.973 DSC.
Mostafa et al. [38]	2015	Cluster	ABC optimization	Fully	2D	Private	93.73% accuracy; 84.82% average SI.
Dou et al. [39]	2016	NN	3D DSN	Fully	3D	SLiver07	5.42% VOE; 0.79 mm ASSD.
Christ et al. [14]	2017	NN	CFCN	Fully	2D	3D-IRCADb01	94.3% mean DSC.
Hiraman [40]	2018	NN	CNN	Fully	2D	SLiver07	12.07% average VOE; −1.96% RVD; 2.25 mm ASSD; 2.60 mm RMSD; 43.01 mm MSSD.
Wang et al. [41]	2019	NN	CNN	Fully	3D	Private	0.94±0.06 DSC.
Almotairi et al. [42]	2020	NN	SegNet	Fully	3D	3D-IRCADb01	94.57% overall accuracy.
Ayalew et al. [44]	2021	NN	U-Net	Fully	2D	3D-IRCADb01; LiTS	0.9612 DSC.
Scicluna [46]	2022	NN	UNet; VGG16UNetC	Fully	2D	CHAOS	85.84 mean score; 97.85 DSC; 80.33 RAVD; 94.80 ASSD score; 70.38 MSSD.
Ezzat et al. [48]	2023	NN	CNN	Fully	2D	Private	98.80% accuracy.
Shao et al. [49]	2024	NN	AC-Net	Fully	3D	Private; LiTS	0.90 DSC; 0.82 JC; 0.92 recall; 0.89 precision; 11.96 HD; 4.59 ASSD.

ABC: Artificial Bee Colony; AC-Net: Attention Connect Network; ASSD: average symmetric surface distance; CFCN: cascaded fully CNN; CNN: convolutional neuronal network; DSC: Dice Similarity Coefficient; DSN: deeply supervised network; GVF: gradient vector flow; HD: Hausdorff distance; JC: Jaccard coefficient; MSSD: maximum symmetric surface distance; NN: neuronal network; RAVD: relative absolute volume difference; RMS: root mean square; RMSD: root mean square Symmetric Surface Distance; RVD: relative volume difference; SI: similarity index; std: standard deviation; VOE: volumetric overlap error.

**Table 2:** Comparison of segmentation categories.

Category	Description	Main Advantages	Main Limitations	Applicability
Threshold	Segments based on intensity thresholds	Simple, fast, easy to implement	Sensitivity to threshold selection, suffers from noise and artefacts	Commonly used in cases where clear intensity differences between the ROI and other regions exist
Edge	Segments based on intensity transitions between regions	Accurate delineation of organ boundaries and structures	Sensitive to noise, difficulties with capturing complex structures	Suitable for images with clear organ boundaries and well-defined edges, but may struggle with low-contrast areas
Region	Segments based on homogeneous regions within an image	Simple implementation, intuitive methodology	Sensitive to initialisation	Often used in cases where interpretability is a concern, but may struggle with fine details
Semantic	Segments based on semantic meaning of pixels	Pixel-level segmentation, fine-grained structural detail	Complex to implement, resource-intensive, and computationally expensive	Suitable for segmenting anatomical structures with distinct features
Cluster	Segments based on similar data patterns or clusters	Efficient grouping and identification of similar data patterns	Sensitivity to initialisation and noise, limited to specific data distributions	Useful for identifying patterns and groups within the data, but can struggle with irregular shapes
NN	Learns models to segment images based on learned features	High accuracy, efficient learning from data	Requires large training datasets, computationally intensive	Suitable for different types of data due to its flexibility and adaptability

**Table 3:** Characteristics of each dataset used by the main analysed studies (based on [61]).

Dataset	Date	Format	Number of Subjects	Slices per Subject	Resolution
MICCAI-SLiver07 [62]	2007	RAW	30	74 to 260	512 × 512
3D-IRCADb01 [43]	2010	DICOM	20	74 to 260	512 × 512
LiTS17 [45]	2017	RAW	200	42 to 1024	Variable
